# *Streptococcus pneumoniae* Invades Endothelial Host Cells via Multiple Pathways and Is Killed in a Lysosome Dependent Manner

**DOI:** 10.1371/journal.pone.0065626

**Published:** 2013-06-13

**Authors:** Henrik Gradstedt, Federico Iovino, Jetta J. E. Bijlsma

**Affiliations:** Laboratory of Molecular Bacteriology, Department of Medical Microbiology, University of Groningen, University Medical Center Groningen (UMCG), Groningen, The Netherlands; Charité-University Medicine Berlin, Germany

## Abstract

*Streptococcus pneumoniae* is one of the major causative agents of pneumonia, sepsis, meningitis and other morbidities. In spite of its heavy disease burden, surprisingly little is known about the mechanisms involved in the switch of life style, from commensal colonizer of the nasopharynx to invasive pathogen. *In vitro* experiments, and mouse models have shown that *S. pneumoniae* can be internalized by host cells, which coupled with intracellular vesicle transport through the cells, *i.e.* transcytosis, is suggested to be the first step of invasive disease. To further dissect the process of *S. pneumoniae* internalization, we chemically inhibited discrete parts of the cellular uptake system. We show that this invasion of the host cells was facilitated via both clathrin- and caveolae-mediated endocytosis. After internalization we demonstrated that the bulk of the internalized *S. pneumoniae* was killed in the lysosome. Interestingly, inhibition of the lysosome altered transcytosis dynamics as it resulted in an increase in the transport of the internalized bacteria out of the cells via the basal side. These results show that uptake of *S. pneumoniae* into host cells occurs via multiple pathways, as opposed to the often proposed view of invasion being dependent on specific, and singular receptor-mediated endocytosis. This indicates that the endothelium not only has a critical role as a physical barrier against *S. pneumoniae* in the blood stream, but also in degrading *S. pneumonia* cells that have adhered to, and invaded the endothelial cells.

## Introduction

*S. pneumoniae* is a Gram-positive bacterium, and commensal colonizer of the nasopharyngeal cavity. Colonization is usually asymptomatic, resulting in clearing of the bacterium. However, *S. pneumoniae* can turn invasive and cause serious disease, such as pneumonia, meningitis, sepsis, and other morbidities. The risk groups include children, elderly, and the immunocompromised. Nasopharyngeal colonization varies, but is markedly higher in young children, approximately 50% at the age of 3 years, and then declines to some 10% after the age of 10 [1].

Given the disease burden caused by this organism, surprisingly little is known about the underlying host-pathogen interactions that govern the switch of *S. pneumoniae* from extracellular commensal colonizer of the nasopharynx to invasive pathogen. Cellular barriers encountered by *S. pneumoniae* before causing invasive disease often contain endothelial cells. For instance *S. pneumoniae* interacts with endothelial cells in the lungs before causing bacteremia and with the blood brain barrier before causing meningitis. Internalization into, and subsequent translocation through the host cells, has been proposed as one possible route of breaching the epithelial and endothelial cell layers that act as barriers against bacteria, *e.g.* the blood brain barrier [2], [3]. Several bacterial factors (*e.g.* PavA, NanA, CbpA, also known as PspC, and) and host cell receptors (*e.g.* PIG-r, PAF-r, and laminin-r) have been shown to be involved in this process [3]–[6]. However, the scope of host factors that mediate *S. pneumoniae* entry into endothelial cells is far from clear.

Here, we have studied the contribution of the cellular endocytosis mechanisms, in particular clathrin- and caveolae-mediated endocytosis in the *S. pneumoniae* invasion of the endothelial host cell. Clathrin- and caveolae-mediated endocytosis are the two main cellular uptake routes, involved in a multitude of cellular endocytosis events. Clathrin endocytosis is dependent on the formation of a coated pit via polymerization of the clathrin tri-skeleton molecule, whereas caveolae-mediated endocytosis is dependent on the presence of cholesterol for proper invagination of the plasma membrane [7]. Both of these uptake mechanisms are used by various bacteria and viruses to gain entry to the host cells. For instance, caveolae-mediated endocytosis is used by *Brucella abortus* and *Streptococcus pyogenes*, closely related to *S. pneumoniae*, to gain entry to the cell [8]. In contrast, clathrin-mediated endocytosis, is used by *Listeria monocytogenes* [7], and it has previously been demonstrated that *S. pneumoniae* can invade transfected COS (African Green Monkey Kidney) cells via clathrin-mediated endocytosis [9], suggesting that this might also be a mechanism of entry for pneumococci in physiologically more relevant cells. After internalization, *S. pneumoniae* can encounter three separate fates: it can be transported to the lysosome for probable degradation, it can be recycled out of the apical (or sidewise) side of the cell [9], or it can translocate through the cell and emerge on the basal side of the host cell [2], facilitating further dissemination into the host. Indeed, some of these internalized *S. pneumoniae* have previously been shown to be associated with various intracellular markers, such as endosomal and lysosomal markers [9], indicating that *S. pneumoniae* is indeed being actively transported in the cell.

To further the understanding of *S. pneumoniae* invasion of endothelial cells, we investigated the contributions of both clathrin- and caveolae-mediated uptake. As *S. pneumoniae* has been shown to associate with lysosomal markers [9], we also investigated the ability of endothelial cells to kill *S. pneumoniae*. Here we show that uptake of *S. pneumoniae* into host cells occurs via multiple pathways, but that the bulk of bacteria are degraded in the lysosome. In conclusion, our study sheds lights on various aspects of *S. pneumoniae* life and death inside host cells. This balance may ultimately govern the traversal of the cellular barriers and further dissemination within the host.

## Materials and Methods

### Bacterial Strains and Growth Conditions

The *S. pneumoniae* strain TIGR4Δ*cps* [10] a capsule-less derivative of strain TIGR4 was used for all experiments. Capsule impedes adhesion and invasion into host cells. However, it has been shown that *S. pneumoniae* in close interaction with host cells loses its capsule [11]. This is why we, like others in the field [2], [9], [11] used uncapsulated bacteria to study the *S. pneumoniae* host cell interactions to increase efficiency of adhesion and invasion. *S. pneumoniae* was routinely grown in M17 broth (Oxoid) supplemented with 0.5% glucose, or on blood agar plates (Mediaproducts bv). Cultures were incubated in a 5% CO_2_ incubator at 37°C or standing in a water bath at 37°C. For start inoculations in all experiments, *S. pneumoniae* aliquots were used. The aliquots were made by growing *S. pneumoniae* in GM17 to a 600 nm optical density of ≈ 0.25, mixed to a 11% glycerol concentration and then frozen in 1 mL aliquots at −80°C.

### Cell Lines and Culture Conditions

Human Brain Microvascular Endothelial Cell (HBMEC) [12] (kind gift from Dr. K.S. Kim) were cultivated in RPMI-1640 medium supplemented with 10% FCS (1% during infection assays, infection assay medium, Biochrom ), 10% Nu-Serum (BD Biosciences), 2 mM L-glutamine, 1 mM sodium pyruvate, 1% minimal essential medium (MEM)-vitamins, and 1% non-essential amino acids (all from Gibco), at 5% CO_2_, 37°C. Fully confluent HBMEC were split once every two or three days via Trypsin/EDTA treatment and diluted in fresh media. The cells were cultivated in tissue culture flasks (TPP#90025) until passage 36.

### Host-pathogen Studies

Prior to infection, confluent HBMEC monolayers in 6 or 12 well plates (TTP 92406 or 92412) were washed repeatedly with RPMI and incubated for 1 h in infection assay medium. Subsequently, ∼5*10∧6 CFU of *S. pneumoniae* were added to each well and incubated for 2 h. Bacterial growth was quantified by sampling the incubation media for non-adherent bacteria prior to their removal by repeated washing with RPMI. To assess *S. pneumoniae* adhesion to host cells, the host cells were lysed with a 50/50 mix of 1% saponin and trypsin-EDTA (0.05%–0.02%). Colony forming units were determined by plating serial dilutions on blood agar plates. To assess invasion of the host cells, any remaining extracellular *S. pneumoniae* cells were eradicated by a 1-h incubation with medium supplemented with gentamycin (50 µg/ml) and penicillin G (2.5 µg/ml), then washed repeatedly with RPMI, lysed and colony forming units were determined as described above. To assess intracellular survival, cells were infected and treated with gentamycin and penicillin G as described above, after which cells were washed repeatedly with RPMI, and subsequently fresh medium containing gentamycin (13.34 µg/ml), penicillin G (0.67 µg/ml) and inhibitors were added at phenotypically non-stressful concentrations. To this end, the host cells were monitored microscopically for stress symptoms, and all inhibitors and bacteria were used at non-stressful concentrations throughout the assays. Three hours after the addition of inhibitors, the cells were lysed and plated as described above.

For translocation assays, transwells with a 3 µm pore size (Costar #3402) were seeded ∼ 5*10^4^ cells/well with HBMEC and grown to confluency. Integrity of the monolayers in the transwells was tested by adding 400 µL HBMEC-HIA +10 ng/ml TNFα to the upper compartment, and waiting for 5 minutes. When no medium appeared in the lower compartment, the monolayer was considered confluent. 1.5 mL HBMEC-HIA +10 ng/ml TNFα was added to the lower compartment and 100 µL media containing 5*10^6^–1*10^7^ CFU’s of *S. pneumoniae* (first washed with HBMEC-HIA+TNFα) was added to the upper compartment. The cells were incubated 2 h at 37°C then gently washed 3× with RPMI. 1 well/strain was used for the adhesion assay (see protocol for adhesion). The remaining wells were switched to new 12 well plates. HBMEC-HIA containing 200 µg/ml gentamycin and 10 µg/ml Penicillin G was added to the upper and lower compartments, and then incubated for 1 h. The wells were then washed 3× with RPMI and 1 well/strain was used for invasion assay (see protocol for invasion). 1.5 and 0.5 ml HBMEC-HIA was added to the lower and upper compartments, respectively. At 3 separate time points (1, 3 & 5 hours) all medium was removed and plated out for CFU determination and new medium was added to both lower and upper compartments.

To determine the integrity of the monolayer during the translocation step a control experiment was performed where approximately 1000 CFU of chloramphenicol resistant *S. pneumoniae* cells were added to the top well after the 3 h of translocation sampling, and their progression to the bottom well was monitored for 2 h by plating out the bottom and top well media on chloramphenicol blood agar plates. Time points 0, 15 min, 30 min, 1 h showed 0 CFU in the bottom well and the 2 h time point showed 0 and 4 CFUs in the bottom well.

### Generation of an Antipneumococcal Antiserum

The *S. pneumoniae* strains TIGR4, D39, and G54 with and without capsule were grown in GM17 to mid-log phase (≈ 0,25 OD_600_), centrifuged, resuspended in PBS, heat inactivated at 100°C for 15 minutes, mixed together, kept at –20°C until used for the inoculation of rabbits according to the Eurogentec (Kaneka Corporation, Osaka, Japan) 3 month antibody production program.

### Confocal Microscopy

Prior to infection, HBMEC cells were seeded and grown to sub-confluency on microscope cover slides in 12 well plates (TTP 92412). Subsequently, ∼ 5*10^6^ CFU of *S. pneumoniae* cells were added to each well and incubated for 2 h, the supernatant removed, fresh media added and incubated for 2 h. The cells were washed repeatedly, then fixed with 3.7% formaldehyde for 15 min at 37°C 5% CO_2_, and blocked with 3% BSA for 45 min. After this the cells were incubated with 1° rabbit–anti-*S. pneumoniae* antibodies for 1 h, then 2° Alexa-Goat-anti-rabbit-350 (Invitrogen A11046) antibody solution. After washing, the cells were permeabilized with 1% triton for 15 min, and blocked with 3% BSA for 45 minutes. The cells were then simultaneously incubated with mouse-anti-CD63 (Sanquin M1544) and rabbit anti-*S. pneumoniae* as primary antibodies for 1 h. Then they were simultaneously stained for 1 h using Alexa-Goat-anti-rabbit-488 (Invitrogen A11008), and Alexa-Goat-anti-mouse-594 (Invitrogen A11005) antibodies. Then the cells were fixed with 0.5 mL 3.7% formaldehyde for 10 min, and mounted on microscope slides. The samples were kept at –20°C until usage. This staining procedure results in blue extracellular *S. pneumoniae*, green intracellular *S. pneumoniae*, and red CD63 in the host cells. Of each cell with clear intracellular *S. pneumoniae,* individual pictures, and Z-stack series (green, red, and grey phase contrast) were taken with a Leica DM IRE2 inverted confocal microscope setup, and subsequently analyzed with ImageJ and its co-localization plug-in function (http://rsb.info.nih.gov/ij/). For the representative image a XY plane merger was created as well as 2 orthogonal views of the YZ and XZ plane [13] were also generated with ImageJ for illustrative purposes.

### Chemicals

The indicated concentrations were selected after titrating out non-stressful concentrations. Their respective solvents were used as controls. For endocytosis inhibition the following endpoint concentrations were used: amantadine (Sigma A1260) 0.5 mM in H_2_O, sucrose (Sigma S0389) 0.2 M in H_2_O, PMA (Sigma P8139) 1 nM in ethanol, Nystatin (Sigma N4014) 20 µg/mL in ethanol, and chlorpromazine (Sigma C8138) 10 µg/mL in H_2_O. All chemicals were added directly after non-adhered bacteria had been washed away. For lysosomal inhibition the following chemicals at the following end concentrations were used: Chloroquione (Sigma C6628) 1,25 µM in H_2_O, and NH4Cl 12,5 mM in H_2_O. Ammonium chloride was added directly after the antibiotics had been washed away to assess invasion, pretreatment of HBMEC cells with chloroquione was performed for ∼ 9 h.

### Statistical Analysis

The SPSS-16 2-tailed Independent-Samples T-test was used to determine significance of the differences obtained with and without inhibitor treatment, presented as p in the results.

## Results

### *S. pneumoniae* can Invade Host Cells via Both Clathrin- and Caveolae-mediated Endocytosis

To determine whether *S. pneumoniae* invades cells via clathrin- or caveolae-mediated endocytosis, we used various well-established chemical inhibitors to block discrete parts of the endocytosis uptake route. One point of concern when employing chemical inhibitors, genetic inhibitors, or markers is that some of these may, or may not, interfere with other cellular functions. To minimize this risk, we used multiple different chemical inhibitors where possible. Additionally, we did not extensively pretreat the cells with inhibitors (except for chloroquine treatment during intracellular survival assays) as this could also create artifacts, but added them during the particular aspect of *S. pneumoniae* and host cell interactions that we were studying.

**Figure 1:**
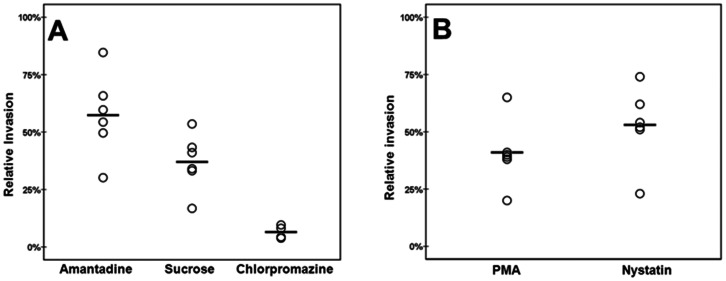
*S. pneumoniae* invasion of endothelial cells is mediated by clathrin and caveolae dependent mechanisms. Invasion in the presence of the vehicle of the inhibitor is set at 100%, and invasion in experimental conditions is related to this percentage. O designates the relative invasion in one individual experiment, ― is the average of all experiments. A: HBMEC cells treated with clathrin mediated endocytosis inhibitors Amantadine, Sucrose, and, Chlorpromazine (n = 6, 6 and 4 respectively). B: HBMEC treated with caveolae- mediated endocytosis inhibitors PMA, and Nystatin (n = 6 and 6). p.<0.05 in all experiments. Invasion is defined as the number of invading bacteria divided by adhered bacteria.

Treatment of the endothelial cells with the clathrin-mediated endocytosis inhibitors amantadine, sucrose, or chlorpromazine [14]–[17] significantly inhibited *S. pneumoniae* invasion (Fig. 1A). Although there was some variation in the reduction of invasion mediated by each inhibitor (amantadine on average 57%, sucrose on average 37%, and, chlorpromazine 6% respectively, compared to the 100% no inhibitor control), the effects were statistically significant (p. <0.05 for all, n = 6, 6, and 4 respectively) in each case compared to the control to which only the solvent of the inhibitors was added. When utilizing the caveolae-mediated endocytosis inhibitors PMA and Nystatin [18], a similarly significant and substantial reduction was observed (on average a 41% and 53% respectively compared to the 100% no inhibitor condition, p. <0.05, n = 6 for both), as can be seen in figure 1B. Combined these results clearly demonstrated that *S. pneumoniae* utilizes both clathrin- and caveolae-mediated endocytosis to gain entry into the endothelial host cell.

### The Bulk of Internalized *S. pneumoniae* is Rapidly Degraded in the Lysosome, but a Minority Survives Longer

**Figure 2:**
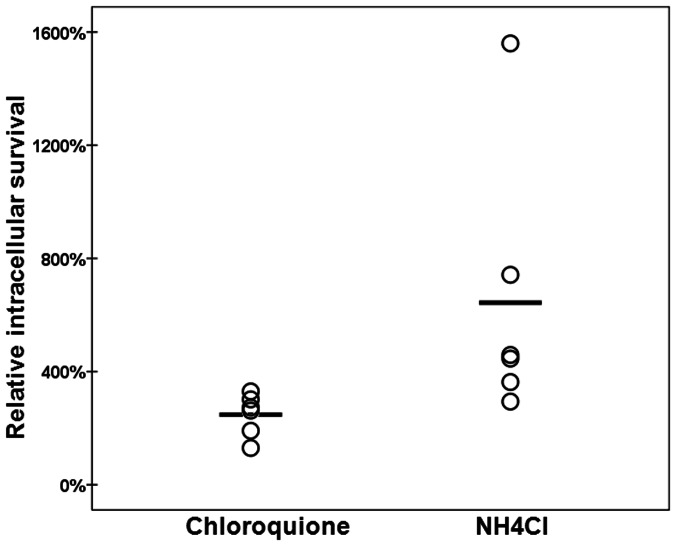
Relative survival of internalized *S. pneumoniae* during inhibition of lysosomal function with chloroquine or NH_4_Cl. Survival in the presence of the vehicle of the inhibitor is set at 100%, and survival in experimental conditions is related to this percentage. O designates the relative survival in one individual experiment, ― is the average of all experiments. (n = 6, and, 6 respectively). p.<0.05 in all experiments. Intracellular survival is defined as number of bacteria obtained after 3 hours of intracellular incubation divided by the number of bacteria obtained after initial invasion.

The “normal”, or at least desirable (from the host cell point of view) fate of a microorganism invading the endothelial host cell is to be degraded in the lysosome. Indeed, we determined that of all invading *S. pneumoniae*, approximately only 1 out of 10 CFU’s were viable after 3 hours inside the host cell, indicating that approximately 90% percent of all intracellular *S. pneumoniae* are killed within the first 3 hours (data not shown). To determine how this dramatic killing of intracellular *S. pneumoniae* occurs, we subjected the endothelial host cells to the well-established lysosomal acidification or maturation inhibitors chloroquine, and NH_4_Cl [19], [20]. As can be seen in figure 2, inhibition of lysosomal function after the bacteria have entered the cells resulted in a significant increase in *S. pneumoniae* intracellular survival (248%, and 643% respectively compared to the 100% no inhibitor control, p. <0.05, n = 6 for both compounds) This clearly shows that most internalized *S. pneumoniae* end up in the lysosome where they are destroyed.

### Intracellular *S. pneumoniae* are Co-localized with CD63

**Figure 3:**
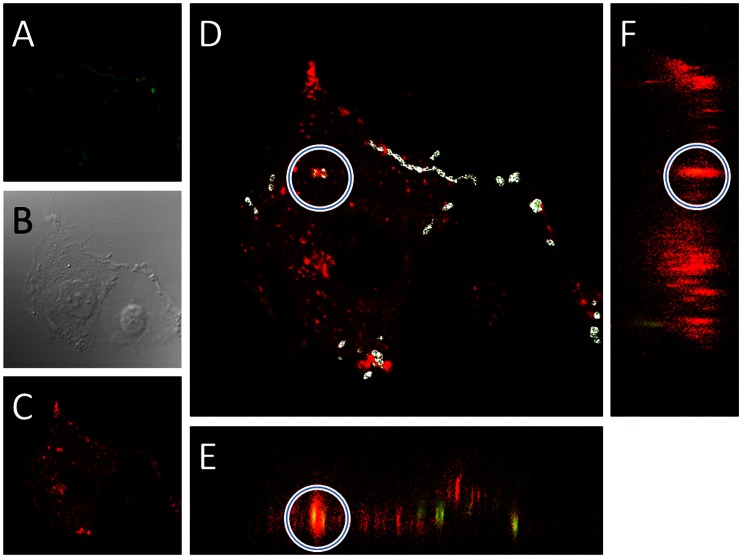
Confocal example of intracellular *S. pneumoniae* colocalized with CD63 in endothelial cells. A: Green *S. pneumoniae* (Alexa-488), B: Phasecontrast, C: Red CD63 (Alexa-594). D: A merge of green and red with a circle around the intracellular *S. pneumoniae* (see results for how this was determined), and the sites of colocalization in white, as identified with the ImageJ Colocalization function. The ImageJ orthogonal view function was used to generate the XZ plane (E) and YZ plane (F) and the site of colocalization between CD36 and intracellular *S. pneumoniae* is seen in yellowish (red and green combined) as highlighted with the white circle. The combined results from image D, E, and F clearly shows that the internalized bacteria are co-localized with the lysosomal marker CD63.

To visualize the intracellular *S. pneumoniae* and lysosome association, we subjected HBMEC cells infected with *S. pneumoniae* to confocal microscopy where we stained for CD63, which is a marker for the plasma membrane and lysosomes [21]. To determine whether *S. pneumoniae* were truly inside the host cell (and not merely tightly associated), we adapted a permeabilisation approach described previously [22], [23]. Intracellular bacteria were first stained with an anti-pneumococcal antiserum, followed by detection with an Alexa 350 (blue) labeled secondary antibody. Subsequently, after selective permeabilization, bacteria were again detected using the antipneumococcal antiserum, but this time an Alexa 488 (green) labeled secondary antibody was used. Intracellular bacteria were then identified by searching for cells that contained bacteria that were only green and had no blue staining. Of such cells, ∼100 sequential Z-stack pictures were taken, starting from the top of the cell and proceeding through to the bottom of the cell. These measures were necessary to provide an unambiguous answer to the extra-, vs. intracellular localization of *S. pneumoniae*, as can be seen in figure 3E–F. In 86% (18 of 21 cells) of cells, which clearly had intracellular *S. pneumoniae*, the bacteria colocalized with CD63 as can be seen in figure 3D–F. Furthermore bacteria tightly adhered to the surface of the endothelial cells also, to a large degree, colocalized with CD63 suggesting that CD63 might be recruited to *S. pneumoniae*-containing vesicles early in the invasion process. These results clearly indicate that the normal fate of internalized *S. pneumoniae* is to be transported to, and degraded in the lysosome via CD63 positive vesicles. Furthermore it highlights the often overlooked ability of the endothelial cells to efficiently kill internalized *S. pneumoniae*.

### Inhibition of the Lysosome Increases *S. pneumoniae* Translocation through Endothelial Cells

**Figure 4:**
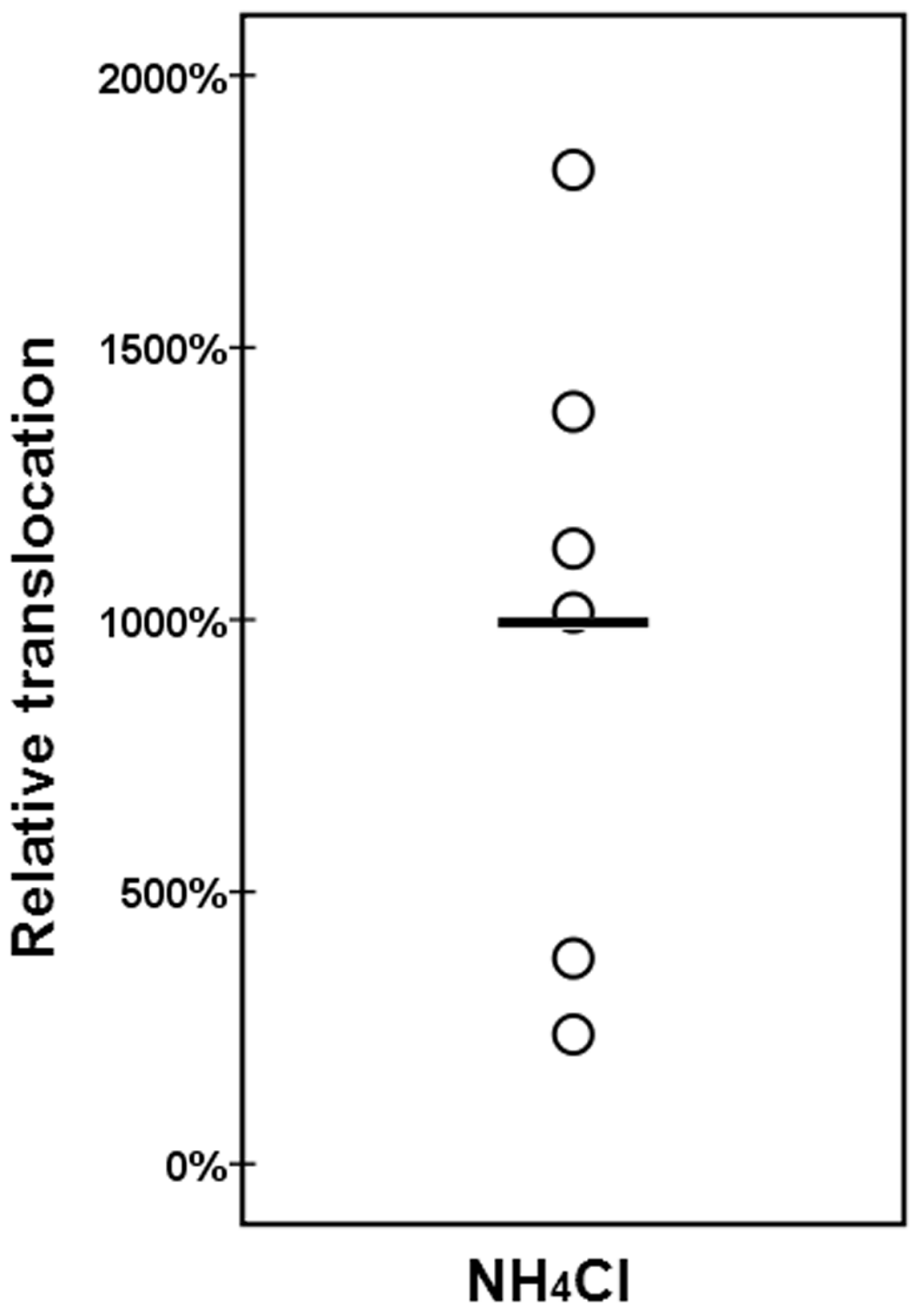
Relative translocation of internalized *S. pneumoniae* when the lysosomal function is inhibited with NH_4_Cl. Translocation in the presence of the vehicle of the inhibitor is set at 100%, and translocation in experimental conditions is related to this percentage. O designates the relative invasion in one individual experiment, ― is the average of all experiments. (n = 6), p.<0.05. Translocation was defined as the cumulative number of bacteria obtained in the lower compartment over 5 hours divided by the number of bacteria obtained at initial invasion point.

To determine whether *S. pneumoniae* transcytosis is a dynamic process, we determined the translocation efficiency of *S. pneumoniae* through endothelial cells when the lysosome was inhibited. As can be seen in figure 4, when the lysosomal maturation inhibitor NH_4_Cl was added directly after *S. pneumoniae* had invaded the endothelial cells, the translocation of *S. pneumoniae* from the apical side, through the cell, to the basolateral side, was increased 10-fold (total translocation CFU/well, on average control ∼ 3.000, NH_4_Cl ∼ 30.000) compared to the control. Combined with the intracellular survival inhibition experiments (Fig. 2), this strongly suggests that a fully functional lysosome system is of paramount importance for the killing of internalized *S. pneumoniae*. Conversely, if the lysosome is impaired, translocation and further dissemination is greatly increased.

## Discussion

In this work, we studied the pathways that *S. pneumoniae* utilizes to enter, survive in, and translocate through endothelial cells, which is thought to be an important part of their capacity to switch from commensal colonizer of the nasopharynx to invasive disease-causing pathogen. Due to our rigorous experimental set up, and the finding that the results of each set of specific inhibitors were very similar, we are convinced that the identified mechanisms are indeed involved in *S. pneumoniae*-host cell interactions, even though any individual methodological approach may cause secondary effects.

Here we have shown that *S. pneumoniae* can utilize clathrin-mediated endocytosis to gain access to the endothelial host cell, which was previously indicated by the finding that in transfected COS (African Green Monkey Kidney) cells treatment with chlorpromazine also inhibited *S. pneumoniae* entry, although not to the same extent as we observed in endothelial cells. [9]. Additionally, the known receptor for *S. pneumoniae* on epithelial cells, PIG-r [3], [4] has been shown to colocalize with clathrin in rat hepatocytes and canine kidney epithelial cells [24], [25], suggesting that clathrin-mediated uptake may also function as entry pathway in epithelial cells. Typically it is estimated that clathrin coated pits can take up particles of approximately 150 nM and it was unclear how larger particle such as bacteria were taken up via this mechanism. Recently it was shown that clathrin also function as an organizer of actin and mediate the uptake of larger particles [26]. Previously, it was also shown that uptake of *S. pneumoniae* depends on actin [2], suggesting that this clathrin-actin pathway is mediating pneumococcal uptake and not the classical clathrin coated pits. In addition to clathrin mediated uptake many bacteria also utilize caveolae mediated endocytosis for invasion of host cells. *Escherichia coli* for instance modulates the actin cytoskeleton into lamellipodia formation which is then taken up by the host cell, at least to some extent via caveolae mediated endocytosis [27]. Here we show, for the first time, that *S. pneumoniae* can also utilize caveolae-dependent endocytosis to invade the endothelial host cell. This has also been shown for some strains of the closely related *S. pyogenes*, were the caveolae-mediated uptake seemed to be mediated by a specific streptococcal protein, SbfI, and the bacteria were associated with caveolae and the protein caveolin1 [8]. This suggests that *S. pneumoniae* may also be associated with caveolin1, and that there might be particular pneumococcal proteins mediating uptake via this pathway. The involvement of caveolae in pneumococcal entry is exceedingly interesting as previous research has indicated that caveolae-mediated uptake may to some extent lead to an evasion of the endosome to lysosome route [7], [8]. This could mean that the *S. pneumoniae* that enter the host cell via caveolae-mediated endocytosis would survive longer than those entering via clathrin-mediated endocytosis. Thus, these particular bacteria might have an increased translocation frequency, possibly resulting in increased virulence.

The fact that *S. pneumoniae* utilizes both clathrin- and caveolae-mediated endocytosis might mean that the internalization process is more dependent on the underlying functions and uptake mechanisms, than on specific receptors. This is a possibility, which should be investigated further. The notion that *S. pneumoniae* can utilize both clathrin- and caveolae-mediated endocytosis to invade the host cell is circumstantially supported in literature. For instance, when looking at signaling pathway activation, the GTPase CDC42 has been shown to be required for efficient *S. pneumoniae* invasion in epithelial cells [28]. When examining the upstream events in CDC42 signaling from other cell types, we find that CDC42 is required/involved in both clathrin- and caveolae-mediated endocytosis (as well as many other cellular functions). However, far more research is needed from the cell biology signaling point of view before any advanced extrapolations can be made as to which signaling pathways directly regulate certain aspects of *S. pneumoniae* interactions with the host cell. Additionally, other studies have indicated the involvement of various signaling pathways in *S. pneumoniae* uptake, whose precise connection with either clathrin- or caveolae-mediated uptake is currently unclear [23], [28], [29].

After invasion, we showed that the standard fate of internalized *S. pneumoniae* is degradation in the endothelial lysosome as was indicated before [9]. This has also been shown in other organisms, for instance *Staphylococcus aureus* and *Legionella pneumophila* [30], [31]. We demonstrated this via both chemical inhibition of lysosomal function, which resulted in substantially increased intracellular survival, and via confocal microscopy, showing a clear colocalization of *S. pneumoniae* and the lysosome. Furthermore, the fact that confocal microscopy unambiguously showed the same results as the usage of chemical inhibitors (*i.e.* that the majority of *S. pneumoniae* is transported to the lysosome) further strengthens the rationale of using chemical inhibitors to dissect the *S. pneumoniae* invasion process.

A minor subset of internalized *S. pneumoniae* cells are most likely recycled out of the cell again. *S. pneumoniae* has been shown to associate with Rab markers, usually associated with exocytosis or ER transport [2], [9]. Yet another subset of the internalized *S. pneumoniae* are neither killed, nor recycled, but translocated from the apical side, through the cell, to the basolateral side [2], [3], [9], [32]. Interestingly, we have shown that this transcytosis process is dynamic and can be altered by inhibiting the lysosome, indicating that there are certain factors and transport pathways that determine *S. pneumoniae* degradation or translocation. Having overcome this cellular line of defense these *S. pneumoniae* would then be “free” to further disseminate throughout the body of the host.

In conclusion, the results presented here clearly indicate that *S. pneumoniae* can gain access to endothelial cells via both clathrin- and caveolae-mediated endocytosis. Once inside the endothelial cell, the greater part of the invading *S. pneumoniae* are degraded in the lysosome. A subset of these internalized *S. pneumoniae* does however avoid lysosomal degradation, and are translocated out of the cell, allowing for further dissemination through the host.
